# Clinical efficacy of antazoline in rapid cardioversion of paroxysmal atrial fibrillation -- a protocol of a single center, randomized, double-blind, placebo-controlled study (the AnPAF Study)

**DOI:** 10.1186/1745-6215-13-162

**Published:** 2012-09-11

**Authors:** Michal M Farkowski, Aleksander Maciag, Rafal Dabrowski, Mariusz Pytkowski, Ilona Kowalik, Hanna Szwed

**Affiliations:** 1 2nd Department of Coronary Artery Disease, 1 Spartanska street, Warsaw, 02-637, Poland

**Keywords:** Antazoline, Atrial fibrillation, Pharmacological cardioversion, Efficacy, Safety, Randomized clinical trial

## Abstract

**Background:**

Rapid conversion of atrial fibrillation (AF) to sinus rhythm may be achieved by the administration of class IA, IC and III antiarrhythmic drugs or vernakalant hydrochloride. However, that treatment may be related to potential pro-arrhythmia, lack of efficacy or the exceptionally high cost of a compound used. Antazoline is a first generation antihistaminic agent with chinidin-like properties. When administered intravenously, antazoline exerts a strong antiarrhythmic effect on supraventricular arrhythmia, especially on AF, facilitating rapid conversion to sinus rhythm. Despite a relative lack of published data antazoline has been marketed in Poland and widely used in cardiology wards and emergency rooms for many years due to its efficacy, safety and rapid onset of action within minutes of administration.

**Methods/design:**

A randomized, double blind, placebo-controlled, superiority clinical trial was designed to assess clinical efficacy of antazoline in rapid conversion of AF to sinus rhythm. Eligible patients will present AF lasting less than 43 hours, will be in stable cardio-pulmonary condition and will have no prior history of advanced heart failure or significant valvular disease. Long-term antiarrhythmic therapy is not considered an exclusion criterion. Subjects who fulfill selection criteria will be randomly assigned to receive intravenously either antazoline or placebo in divided doses and observed for 1.5 hours after conversion to sinus rhythm or after the last i.v. bolus. Primary end point will be the conversion of AF to sinus rhythm confirmed in an electrocardiogram (ECG) during the observation period. Secondary end points will be comprised of time to conversion and return of AF during the observation period. Special consideration will be given to the observation of any adverse events. A sample size of 80 patients was calculated based on the following assumptions: two-tailed test, a type I error of 0.01, a power of 90%, efficacy of placebo 5%, efficacy of antazoline 50% and 20% drop-out rate to fulfill the criteria of intention-to-treat analysis. Due to the presumed lack of statistical power, the secondary end points and safety endpoints will be considered exploratory.

**Clinical trials registry:**

ClinicalTrials.gov, NCT01527279

## Background

Atrial fibrillation (AF) is considered to be not only a medical but also a social problem. It is related to the elevated risk of thrombo-embolic events with a stroke being the most important and it adversely affects life expectancy and quality. The cornerstone of AF therapy is a long-term antithrombotic treatment followed by adequate rhythm or rate control [1,2]. The pharmacological cardioversion (CV) of AF to sinus rhythm (SR) may be achieved by administration of class IA, IC and III antiarrhythmic drugs: flecainide, ibutilide, dofetilide, propafenone or amiodarone [1,2]. Beta-adrenergic blocking agents are not considered suitable for pharmacological CV due to their low or lack of efficacy. Another drug recently introduced for rapid CV of AF is vernakalant [3-6]. Pharmacological CV may be related to potential pro arrhythmia, lack of efficacy or exceptionally high cost of a compound used.

So far there is no significant evidence of superiority of either rate or rhythm control strategies in terms of mortality, severe AF complication rates, worsening of heart failure quality of life of patients with AF. A hypothesis that deleterious effects of antiarrhythmic drugs may have offset the benefits of SR in those patients is seriously considered. Therefore a decision of SR restoration and maintenance for a long time should be patient-tailored and based on the individual patient’s history, symptoms and preferences [1].

Antazoline is a first generation antihistaminic agent with chinidine-like and anticholinergic properties. Antazoline prolongs action potential duration and lowers its amplitude, prolongs phase 0 duration, reduces phase 4 of resting potential and reduces excitability of cardiac tissue. Clinically, antazoline lowers the velocity of intraatrial conduction, prolongs the atrial refraction period and may improve atrioventricular conduction allowing fast ventricular response to supraventricular arrhythmias. The half-life of antazoline is considered to be about three hours with antiarrhythmic efficacy expiring after about one hour [7-9].

There are no widely known sound randomized clinical studies conducted to evaluate the antiarrhythmic effect of antazoline. Published studies were mainly single-arm clinical trials with no control group or a different series of cases where antazoline was administered either orally or intravenously in different doses and in different arrhythmias. These studies suggested high efficacy of antazoline in rapid conversion of AF to SR if administered intravenously up to the cumulated dose of 350 mg. Most adverse effects were observed after cumulated doses exceeding 250 mg and they were mainly comprised of mild hypotension, hot flushes and mild tachycardia. Antazoline can unmask the underlying sick sinus syndrome or atrio-ventricular block [7,8,10-27]. Moreover, antazoline has been used in clinical practice in Poland for many years due to its efficacy, safety and rapid onset of action within minutes of administration. According to the Summary of Product Characteristics**,** antazoline is indicated in the treatment of paroxysmal supraventricular tachyarrhythmias including AF and should be administered intravenously in a cumulative dose of 100 to 300 mg during 3 to 10 minutes under strict monitoring of ECG and arterial blood pressure and interrupted after conversion to SR.

The aim of this randomized, double blind, placebo-controlled, superiority clinical trial is to assess clinical efficacy of antazoline in rapid conversion of atrial fibrillation to sinus rhythm in patients with paroxysmal atrial fibrillation without significant valvular disease or advanced heart failure.

## Methods/design

The study protocol was approved by the local ethics committee and is in full compliance with the Declaration of Helsinki.

### Participants

All patients with AF lasting less than 43 hours reporting to the emergency room (ER) or clinical ward of our center will be considered for inclusion.

### Inclusion criteria

 – Written informed consent for participating in the study and written standard version of informed consent for cardioversion accepted at the Institute of Cardiology, Warsaw, Poland

 – Age 40 to 75 years

 – Potassium blood concentration over 3.5 mmol/l

 – Stable cardio-pulmonary state on enrollment

 – In case of unclear history of heart failure or suspicion of left ventricle damage echocardiography is indicated prior to enrollment

 – A long-term antiarrhythmic drug therapy is allowed

### Exclusion criteria

 – Lack of written informed consent

 – Allergy to antazoline

 – AF related to significant valvular disease

 – Clinically significant heart failure or ejection fraction <55%

 – Diastolic blood pressure (BP) <100 mmHg

 – History of significant bradyarrhythmia not treated with permanent pacemaker

 – QT prolongation over 440 ms or QTc (Bazett’s formula) over the population norm

 – Tachycardia >160’

 – Advanced liver or kidney failure

 – Acute coronary syndrome, coronary artery by-pass graft, stroke or transient ischemic attack within 30 days before enrollment

 – Preexcitation in ECG not treated by radiofrequency ablation of accessory pathway

 – Signs and symptoms of ischemia related to AF

 – An investigational drug used within 30 days before enrollment

 – Pregnancy or breast feeding

### Time window

The AnPAF Study is designed both for symptomatic and asymptomatic patients.

In symptomatic patients the self-reported onset of symptoms is considered when determining the time of AF episode. In symptomatic patients on long term antiarrhythmics or oral anticoagulants the self-reported onset of symptoms of a recent episode of AF is considered when determining the inclusion criteria.

In asymptomatic patients a medical documentation of SR within the last 43 hours must be available to enroll the patient (for example ECG strip).

In order to ensure patients’ safety in all ambiguous or controversial cases patients will not be considered for enrollment.

### Interventions

#### Both groups

Apart from the assigned drug the treatment of both groups will not differ at any time during the study.

Any patient fulfilling the inclusion criteria will be prepared to pharmacological CV in a standard way comprising the standard baseline 12-lead ECG, continuous ECG monitoring, periodic noninvasive blood pressure monitoring (BP) and iv line. The study drug or placebo will be administered intravenously in boluses by a nurse under supervision of the enrolling physician, both blinded to patient assignment. Both substances will be prepared in syringes by the study nurse unblinded to patient assignment. After administration the patient will be observed for 1.5 hour after the last dose with exit ECG and BP measure taken at the end of observation. Further treatment of the patient depends on the clinical state and follows current clinical guidelines.

#### Study group

Patients assigned to the antazoline group will be administered antazoline in boluses of 50 mg diluted to 10 cm^3^ every five minutes up to a cumulative dose of 250 mg or conversion of AF to SR. Drug administration will also be stopped in case of an adverse event (see Outcomes section) or conversion of AF to a different supraventricular arrhythmia. BP will be measured before every injection.

#### Control group

Patients assigned to the control group will be administered 0.9% saline in boluses of 10 cm^3^ every five minutes up to a cumulative volume of 50 cm^3^, conversion of AF to SR or in case of an adverse event (see Outcomes section) or conversion of AF to a different supraventricular arrhythmia. BP will be measured before every injection.

### Objectives

The purpose of the study is to assess clinical efficacy of antazoline in rapid conversion of AF to SR in patients with paroxysmal atrial fibrillation without significant valvular disease or advanced heart failure. Due to a presumed lack of statistical power, secondary end points and safety analysis will be considered exploratory.

### Outcomes

All clinical outcomes will be assessed by the enrolling physician and later adjudicated by an experienced cardiologist both blinded to patients assignment.

All safety outcomes will be assessed by the enrolling physician blinded to patient’s assignment. In order to provide maximum safety for the patient, a physician unblinded to patent’s assignment but not involved in the clinical outcomes assessment will be available at the study site at all times.

**Primary outcome:** conversion of AF to SR confirmed in standard 12-lead ECG during the observation period

**Secondary end points:** time to conversion; return of AF during observation period; serious adverse event defined as **every** adverse event requiring hospitalization or prolonged observation

### Safety endpoints

 – BP <90 mmHg

 – Disturbances of atrio-ventricular conduction

 – Sustained supraventricular arrhythmia other than AF

 – New complex ventricular arrhythmia

 – Hot flush

 – Drowsiness

 – Headache

 – Nausea/ vomiting

 – Chest pain

 – Tachycardia >180’

 – Prolongation of QTc (Bazett’s formula) in comparison to baseline (in ms)

### Randomization

Randomization will be provided by the independent statistician using SAS.9.2 software, SAS Institute Inc., Cary, NC, U.S.A.. Permuted block randomization will be used with a block size (AB, BA) not known by the investigators. After eligible patients give informed consent a specific unique identifier will be assigned to the patients. Use of this randomization process ensures proper trial enrollment and promptly provides the statistician with the basic patients enrollment information needed to monitor enrollment performance.

### Allocation concealment

A Random allocation sequence will be implemented using numbered sealed envelopes opened after inclusion of the patient for the study.

### Implementation

After inclusion of the patient the study the nurse will open the numbered envelope and prepare five 10 cm^3^ syringes with study drug or placebo according to randomization and pass them to the enrolling physician and nurse who will administer the drug.

### Blinding

The study will be conducted in double-blind fashion. The patient, enrolling physician, nurse who administering the drug, and clinician reviewing the clinical outcomes will all be blinded to the treatment. The statistician, study nurse who prepares the syringes and clinician involved in safety control will be unblinded to the patient’s assignment.

## Statistical methods

### Sample size

The clinical efficacy of antazoline was assumed using data derived from Srzednicki *et al.* 1990 [8], the most credible publication available. Assumption of 50% efficacy in AF conversion is conservative, since the authors reported efficacy over 70% in doses not exceeding 250 mg of antazoline. Assumption of 5% efficacy of placebo was derived from studies over vernakalant [3-6]. Using a two-tailed test, a type I error of 0.01, a power of 90% and a 20% drop-out rate to fulfill the criteria of intention-to-treat analysis, we calculated that we need 80 patients, 40 in each group, to show the superiority of antazoline over placebo.

### Data management

During the study, the investigator will regularly enter information into case report forms (CRFs). Database management and quality control for this study will be the responsibility of the Department of Biostatistics (this is an independent statistician). Structured data elements from the CRFs will be entered into the database and reviewed using double data entry for verification. Information entered into the database will be systematically checked and obvious errors will be corrected. Omissions or questions will be returned to the investigator for resolutions.

### Statistical analysis

All analyses will be conducted using SAS software (version 9.2., SAS Institute Inc., Cary, NC, USA. The Kolmgorov-Smirnov test will be used to check for normal distribution of continuous data. Normally distributed continuous data will be presented in terms of mean ± standard deviation. They will be compared across two groups with the two-sided unpaired t-test or Cochran-Cox test when there is heterogeneity of variance (evaluated by the F-Snedocor test). Non-normally distributed continuous data will be reported in terms of percentiles (for example, median and inter-quartile range) and will be compared across two groups by Wilcoxon rank-sum test. Student’s paired *t*- or Wilcoxon’s test will be used as appropriate to compare continuous variable differences between baseline and the end of the observation period. Categorical variables will be summarized in terms of frequencies and percentages. A comparison between them will be performed by Pearson’s χ^2^ test with continuity correction or Fisher’s exact test, if the expected cell count will be less than 5. The probability of return to SR (by the time from AF) by the treatment group will be graphically displayed according to the method of Kaplan–Meier, with comparison of cumulative events by the log-rank test. Multivariate analysis will be carried out using Cox proportional hazards regression modeling. All hypotheses will be two-tailed with a 0.05 type I error.

## Trial status

We are in the recruiting phase.

## Abbreviations

AF: atrial fibrillation; BP: blood pressure; CRFs: case report forms; CV: cardioversion; ECG: electrocardiogram; ER: emergency room; QTc: corrected QT interval; SR: sinus rhythm.

## Competing interests

The study is funded entirely by the Institute of Cardiology, Warsaw, Poland. The authors declare that they have no competing interests.

## Authors’ contributions

All authors contributed to the development of the study protocol and this manuscript. According to the situation, MMF, AM, RD and MP will be acting as consulting physicians or safety supports but not both for the same patient. AM is the project manager. IK is the study statistician. HS is the head of the Steering Committee. All authors read and approved the final manuscript.

## Authors’ information

The study is funded entirely by the Institute of Cardiology, Warsaw, Poland. All authors are employees of the Institute of Cardiology, Warsaw, Poland. There are no other sources for funding of this study.
